# Cost-related medication nonadherence in adults with COPD in the United States 2013–2020

**DOI:** 10.1186/s12889-024-18333-z

**Published:** 2024-03-20

**Authors:** Xin Wen, Hongbin Qiu, Bo Yu, Jinfeng Bi, Xia Gu, Yiying Zhang, Shanjie Wang

**Affiliations:** 1 Department of Epidemiology and Biostatistics, School of Public Health, Jiamusi University, 258 Xuefu Road, Xiangyang District, Jiamusi, 154007 China; 2 Department of Cardiology, Second Affiliated Hospital of Harbin Medical University, 246 Xuefu Road, Nangang District, Harbin, 150086 China; 3 The Key Laboratory of Myocardial Ischemia, Chinese Ministry of Education; National Key Laboratory of Frigid Zone Cardiovascular Diseases, Harbin, China; 4 Department of Respiratory, Second Affiliated Hospital of Harbin Medical University, Harbin, China

**Keywords:** Chronic obstructive pulmonary disease, Cost-related medication adherence, Nonadherence behaviors, Self-management, Vulnerable population

## Abstract

**Background:**

Cost-related medication nonadherence (CRN) is associated with poor prognosis among patients with chronic obstructive pulmonary disease (COPD), a population that requires long-term treatment for secondary prevention. In this study, we aimed to estimate the prevalence and sociodemographic characteristics of CRN in individuals with COPD in the US.

**Methods:**

In a nationally representative survey of US adults in the National Health Interview Survey (2013–2020), we identified individuals aged ≥18 years with a self-reported history of COPD. Cross-sectional study.

**Results:**

Of the 15,928 surveyed individuals, a weighted 18.56% (2.39 million) reported experiencing CRN, including 12.50% (1.61 million) missing doses, 13.30% (1.72 million) taking lower than prescribed doses, and 15.74% (2.03 million) delaying filling prescriptions to save costs. Factors including age < 65 years, female sex, low family income, lack of health insurance, and multimorbidity were associated with CRN.

**Conclusions:**

In the US, one in six adults with COPD reported CRN. The influencing factors of CRN are multifaceted and necessitating more rigorous research. Targeted interventions based on the identified influencing factors in this study are recommended to enhance medication adherence among COPD patients.

**Supplementary Information:**

The online version contains supplementary material available at 10.1186/s12889-024-18333-z.

## Background

Chronic obstructive pulmonary disease (COPD) is a persistent and progressive airway disease characterized by difficulty in breathing, coughing, and sputum production [1–4]. According to the World Health Organization (WHO), COPD is the third leading cause of chronic morbidity and mortality worldwide, including in the US and China [3, 5–8]. In 2017, the estimated number of people living with chronic respiratory diseases worldwide was at 544 million, with approximately 55% of cases attributable to COPD [4, 7].

The Global Initiative on Obstructive Lung Disease (GOLD) recommends that patients with COPD should undergo long-term therapy to improve prognosis, particularly through medication adherence [9, 10]. However, long-term medication adherence in patients with COPD is poor, which is less than 50% according to the WHO reports [11, 12]. Increasing medication nonadherence is associated with increasing COPD symptoms, morbidity, hospitalizations, mortality, and healthcare expenditures [11, 13, 14]. Although medication nonadherence is complex and multifactorial, cost-related medication nonadherence (CRN) is modifiable. Effective drug management has been shown to reduce the financial burden on and improve the clinical characteristics and prognosis of patients [15].

Although previous studies have reported poor long-term medication adherence among patients with COPD, recent data on the prevalence of this issue are lacking. Moreover, there is a paucity of studies about CRN in US COPD patients. Furthermore, previous study had demonstrated that economic disparities and inadequate insurance coverage negatively impact healthcare for individuals with COPD [16]. However, despite efforts such as the Affordable Care Act (ACA), these challenges have not been adequately addressed [16]. Despite expanded insurance coverage, escalating prescription drug costs have resulted in no improvement or even a decline in the affordability of care for individuals with COPD [14, 16]. Therefore, improving medication adherence and health outcomes in patients with COPD remains an urgent and elusive challenge for the global health community [3]. In this study, we used nationally representative data to investigate the prevalence and sociodemographic characteristics of CRN among adults with COPD in the US to provide healthcare systems with recommendations for health interventions to improve patient outcomes and enhance the quality of life of patients with COPD.

## Methods

### Data source

We used data from the National Health Interview Survey (NHIS) obtained over the past 8 years from 2013 to 2020 [17]. The NHIS, conducted annually by the US National Centre of Health Statistics of the Centers for Disease Control and Prevention, is an ongoing cross-sectional national health survey that utilizes a complicated and multistage sampling strategy to provide estimates on the noninstitutionalized US population [17]. As NHIS data are publicly available and deidentified, this study was exempt from approval by the institutional review board.

### Study population and outcome

We included adults aged ≥18 years who were diagnosed with COPD based on a positive response to any of the following questions: (1) “During the past 12 months, have you been told by a doctor or other health professional that you had emphysema?”; (2) “During the past 12 months, have you been told by a doctor or other health professional that you had chronic obstructive pulmonary disease, also called COPD?”; and (3) “During the past 12 months, have you been told by a doctor or other health professional that you had chronic bronchitis?” [18] Of the 15,928 individuals with COPD included in this study, 2881 (17.83%) self-reported CRN.

The primary outcome was CRN, which was defined as a positive answer to any of the following questions: (1) “During the past 12 months, have you skipped medication doses to save money?”; (2) “During the past 12 months, have you taken less medicine than prescribed to save money?”; and (3) “During the past 12 months, have you delayed filling a prescription to save money?” [18–20].

### Statistical analyses

The data collected in the NHIS are obtained through a complex sample design that involves stratification, clustering, and oversampling of specific subpopulations. As recommended by the NHIS, we used the statistical analysis tool to estimate the nationally representative characteristics, including all relevant year-specific stratification, clustering, and weighting parameters [20, 21]. For the multiyear datasets, the sampled individuals vary across the years. Participant-level weights were divided by the number of years to obtain appropriate sampling weights [20, 22]. Descriptive statistical analyses were used to obtain weighted estimates for the proportion of patients with COPD who reported one or more CRN measures. To assess the temporal changes in reported CRN, we examined the trends of these CRN measures over time [19].

The χ^2^ test was used to compare the prevalence of categorical variables among different groups. Considering the variation in access to health insurance coverage among US citizens, the descriptive analyses were stratified according to age < 65 or ≥ 65 years [19]. Furthermore, to evaluate the potential risk factors associated with CRN, including demographic (age, sex, and race/ethnicity), socioeconomic (household income, insurance status, and education), geographic, and morbidities, we constructed multivariate logistic regression models [19]. We included the total number of morbidities (0, 1, and ≥ 2) as predictors in the model [19]. Multivariate logistic regression was used to estimate the odds ratios (ORs) with 95% confidence intervals (CIs).

A sensitivity analysis was performed to verify the robustness. We separately analyzed medication nonadherence in patients with chronic bronchitis or emphysema duo to their distinctive therapeutic principles. Missing values of covariates less than 5% were filled with multiple imputations. All statistical analyses were performed using Stata 15.1 (Stata Corp, College Station, TX). *P* ≤ 0.05 was considered statistically significant.

## Results

Of the 253,577 US adults included in the NHIS from 2013 to 2020, 17,591 reported COPD. This corresponds to 5.9% of US adults, representing an estimated 14.38 million patients per year (95% confidence interval [CI] 14.02–14.78 million). Of the 17,591 adults with COPD, 15,928 (90.54%) completed the individual components for CRN, and 2881 self-reported experience of CRN. This equates to an average of 2.39 million (95% CI 2.28–2.51 million; 18.56%) US adults with COPD reporting CRN each year from 2013 to 2020 (Supplemental Fig. 1).

### Prevalence and risk factors for CRN

**Fig. 1:**
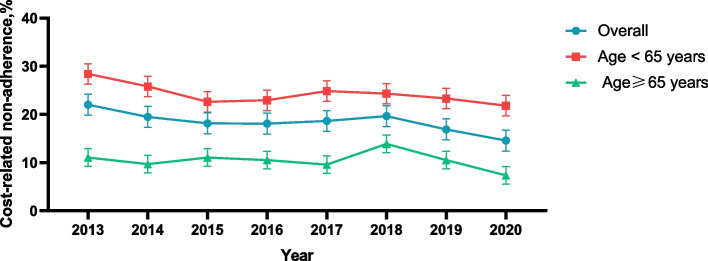
Calendar-year trends in cost-related medication nonadherence. Error bars represent 95% confidence intervals

A total of 1.61 million individuals (1.52–1.71 million; 12.50%) with COPD skipped doses of medication, 1.72 million (1.62–1.81 million; 13.30%) took less medication than prescribed, and 2.03 million (1.93–2.13 million; 15.74%) delayed filling prescriptions to save costs. In addition, average rates of CRN decreased from 22.03% in 2013 to 14.57% in 2020 among individuals with COPD (*P* < 0.01). Stratifying by age, different age groups exhibited a consistent trend similar to the overall population, indicating a decline in CRN prevalence between 2013 and 2020 (< 65: 28.41–21.83%; ≥65:11.04–7.36%) (P < 0.01) (Fig. 1 and Supplemental Fig. 2).

**Fig. 2:**
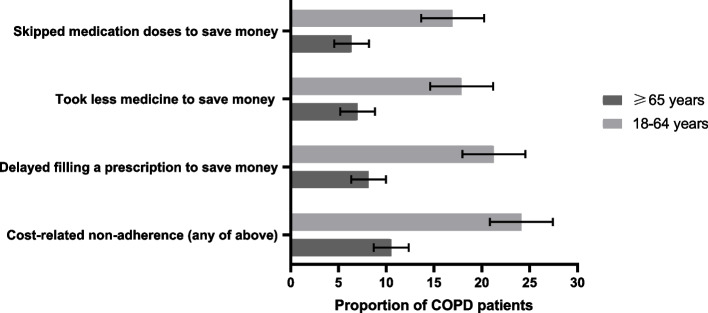
Rates of cost-related medication nonadherence and its components by age groups. COPD indicates chronic obstructive pulmonary disease. Error bars represent 95% confidence intervals

Overall, the prevalence of CRN was higher among patients with COPD who were younger, women, with low family income, uninsured, and greater morbidity burden (Table 1). A total of 24.39% of patients with COPD aged < 65 years reported CRN, whereas only 10.52% of patients with COPD aged ≥65 years reported CRN (Fig. 2). Patients aged < 65 years in certain population segments were particularly prone to report CRN, with 1 in 4 women, 1 in 4 patients from low-income families, and nearly 50% of the uninsured patients reporting CRN (Supplemental Table 1 and 2). Compared with patients with COPD aged ≥65 years, those aged < 65 years were two-fold more likely to refuse prescribed medication to save costs (16.94% vs. 6.38%), take less medication than prescribed (17.87% vs. 7.00%), and delay filling a prescription (21.25% vs. 8.15%) (Fig. 2).

**Table 1:** Characteristics among adults with chronic obstructive pulmonary disease based on whether they reported cost-related nonadherence

Variable	No Cost-Related Nonadherence, Weighted % (95% CI)	Cost-Related Nonadherence, Weighted % (95% CI)	*P*
Sample, n	13,047	2881	
Weighted sample, *n* (weighted %)	10,481,522(81.44)	2,388,717(18.56)	
Age category, y			< 0.001
18–64	53.81(52.62,54.98)	76.18(74.10,78.14)	
≥ 65	46.19(45.02,47.38)	23.82(21.86,25.90)	
Female	58.28(57.11,59.43)	67.68(65.32,69.94)	< 0.001
Race/ethnicity			< 0.001
Non-Hispanic white	78.78(77.56,79.95)	73.9(71.48,76.18)	
Hispanic	7.13(6.38,7.97)	9.46(7.98,11.18)	
Non-Hispanic black	10.54(9.71,11.44)	13.32(11.67,15.16)	
Non-Hispanic Asian	2.00(1.67,2.39)	1.44(0.91,2.25)	
Region			< 0.001
Northeast	16.98(15.56,18.49)	12.75(11.15,14.54)	
Midwest	24.21(22.44,26.07)	25.63(22.99,28.45)	
South	40.56(38.61,42.53)	45.42(42.53,48.35)	
West	18.26(16.73,19.89)	16.20(14.23,18.38)	
Education level			0.013
Less than high school	16.58(15.73,17.46)	19.54(17.71,21.51)	
High school graduate	24.50(23.52,25.50)	23.02(21.07,25.10)	
College or above	58.92(57.70,60.14)	57.44(54.94,59.91)	
Household income			< 0.001
Low	42.26(40.95,43.57)	59.01(56.62,61.35)	
Middle	29.75(28.60,30.93)	27.79(25.67,30.01)	
High	27.99(26.83,29.18)	13.21(11.56,15.05)	
Insurance status			< 0.001
Public	50.32(49.08,51.56)	50.43(48.04,52.82)	
Private	48.22(46.98,49.46)	44.28(41.93,46.66)	
Uninsured	1.46(1.17,1.82)	5.29(4.16,6.70)	
Smoking status			< 0.001
Never	30.67(29.53,31.85)	27.61(25.43,29.90)	
Previous	40.32(39.17,41.49)	29.74(27.61,31.97)	
Current	29.00(27.90,30.13)	42.65(40.19,45.16)	
Alcohol drinking status^a^			0.146
Never	16.33(15.34,17.37)	14.32(12.64,16.17)	
Previous	28.96(27.86,30.09)	29.08(26.76,31.51)	
Current	54.71(53.44,55.97)	56.60(53.93,59.24)	
Morbidities			
Hypertension	61.14(59.94,62.34)	60.68(58.24,63.06)	0.733
High cholesterol	51.43(50.30,52.56)	52.39(49.94,54.84)	0.479
Coronary heart disease	33.70(32.59,34.82)	34.13(31.74,36.60)	0.756
Stroke	10.67(10.00,11.38)	10.48(9.16,11.96)	0.808
Asthma	36.69(35.62,37.77)	47.94(45.57,50.33)	< 0.001
Cancer	21.62(20.75,22.52)	18.64(16.87,20.54)	0.006
Diabetes	22.16(21.25,23.10)	26.10(24.04,28.28)	0.001
Chronic kidney disease^a^	7.83(7.21,8.50)	9.93(8.41,11.69)	0.022
Insufficient physical activity^a^	82.27(80.92,83.54)	83.89(81.05,86.37)	0.269
BMI			< 0.001
Normal (< 25 kg/m^2^)	18.20(17.32,19.13)	16.92(15.14,18.87)	
Overweight (25–30 kg/m^2^)	28.90(27.87,29.94)	24.43(22.48,26.50)	
Obese (> 30 kg/m^2^)	52.90(51.72,54.07)	58.64(56.28,60.97)	

BMI indicates body mass index

Missing values of covariates less than 5% were filled with multiple imputations

^a^Data on alcohol drinking status, chronic kidney disease, and insufficient physical activity were not surveyed in 2019 which were excluded in the corresponding analysis

**Fig. 3:**
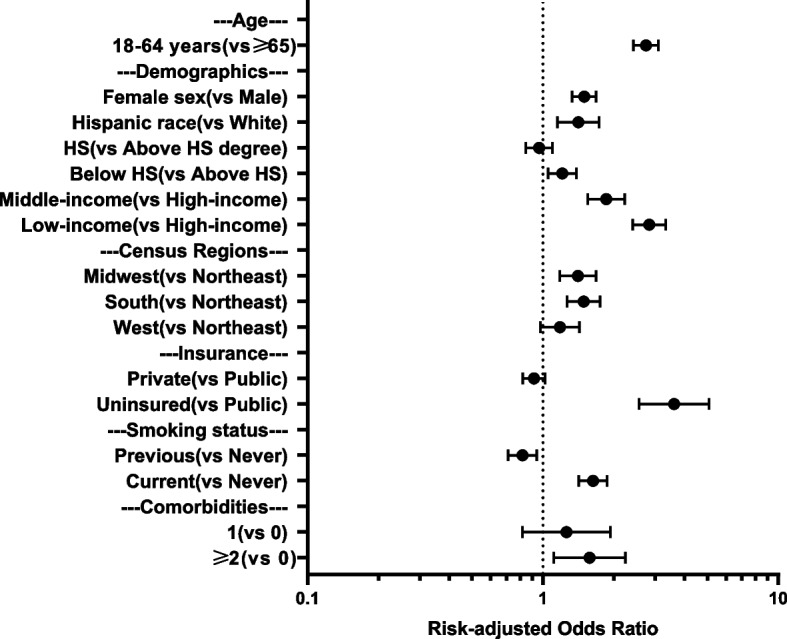
Predictors of cost-related medication nonadherence. HS indicates high school. The number of morbidities was defined as the sum of the following conditions that a respondent was ever told by a doctor or other health professional that he or she had: hypertension, high cholesterol, coronary heart disease, stroke, cancer, diabetes, kidney weak, and insufficient physical activity. Since the analysis results of individual morbidities are consistent with those of the total number of morbidities. Therefore, only the analysis results for the total number of morbidities are presented in Figure. Missing values of covariates less than 5% were filled with multiple imputations. Data on alcohol drinking status, chronic kidney disease, and insufficient physical activity were not surveyed in 2019 which were excluded in the corresponding analysis

In a multivariable logistic regression model that accounted for age, sex, race, education, family income, insurance, and comorbidities, CRN was more likely to occur in younger age groups (18–39, 40–64 years) than in the ≥65 years age group (odds ratio [OR], 2.76 [95% CI, 2.29–3.33]; 2.74 [95% CI, 2.42–3.09]). The factors most strongly associated with reporting CRN included female sex (OR, 1.49 [95% CI, 1.33–1.68]), low family income (OR, 2.83 [95% CI, 2.41–3.32]), lack of insurance (OR, 3.61 [95% CI, 2.56–5.07]), being a current smoker (OR, 1.63 [95% CI, 1.42–1.87]), asthma (OR, 1.59 [95% CI, 1.42–1.76]) and the number of high comorbidities (1 morbidity: OR, 1.25 [95% CI, 0.81–1.93]; ≥2 morbidities: OR, 1.57 [95% CI, 1.11–2.24]) (Fig. 3). Similar factors were also associated with CRN in the older (≥65 years of age) and younger (18–64 years) age groups of patients with COPD (Supplement Table 3).

### Sensitivity analysis

The sensitivity analysis included all patients with Chronic bronchitis and emphysema and analyzed them separately. A total of 18.80 and 22.26% of patients with emphysema and chronic bronchitis, respectively, reported CRN. The prevalence of CRN was higher among young, female, low household income, and uninsured patients with emphysema or chronic bronchitis. The results of the analysis were consistent with those of patients with COPD (Supplemental Tables 4–7).

## Discussion

In this nationally representative study conducted in the US using the most updated NHIS data (2013–2020), we found that nearly one in six individuals with COPD, which equates to 2.39 million adults, was nonadherent with their medications due to medication costs. The prevalence of CRN was higher among younger individuals, women, low-income families, uninsured, and greater morbidity burden, which is consistent with previous reports [23].

Overall, from 2013 to 2020, the rate of reported CRN in patients with COPD had a declining pattern, from 22.03% in 2013 to 14.57% in 2020, likely because of the enactment and implementation of the Patient Protection and ACA in 2014 in the United States, which was designed to increase health insurance coverage and provide low-income populations with making financial subsidies to reduce individual health care costs [16, 24].

Furthermore, our study demonstrated a significant difference in the prevalence of CRN between the < 65 years and ≥ 65 years age groups of individuals with COPD. Although individuals with COPD aged < 65 years were more likely to have fewer comorbidities than those aged ≥65 years, they were twice as likely to not adhere to medication. There are multiple factors contributing to CRN among COPD patients aged < 65 years and ≥ 65 years, encompassing economic aspects such as insurance and drug costs, as well as patient behavior and perception factors [25–28]. The absence of insurance may impact patients’ reporting of CRN. Uninsured individuals might postpone or discontinue treatment due to the exorbitant medical expenses associated with COPD management. However, even with medical insurance coverage, CRN can still arise owing to out-of-pocket (OOP) prescription drug costs [29]. Although insurance partially reimburses these expenses, patients are still required to bear a certain amount OOP which could pose a barrier for those facing financial constraints [5, 29]. Furthermore, younger individuals tend to prioritize personal interests and immediate gratification, they may pay more attention to the short-term effects of drugs and ignore the long-term treatment effects [12]. Consequently, when they fail to perceive the benefits of medication clearly, they might opt not to adhere to medical advice owing to the exorbitant cost associated with pharmaceuticals [12]. Additionally, young individuals often exhibit heightened confidence in their physical well-being and self-healing capabilities while harboring reservations regarding the effectiveness of medications, this skepticism might impact their adherence [12].

Our study revealed that individuals with low household income were more likely to report CRN. In 2016, the total spending on all respiratory illnesses in the US was $170.8 billion, which has increased by $71.7 billion since 1996. In the same year, the respiratory condition with the highest spending was COPD, contributing $34.3 billion [30]. The debilitating properties of COPD, a highly prevalent respiratory disease, place a significant financial burden on the US healthcare system [3, 31]. Furthermore, OOP prescription medication costs are increasing, and are a major factor influencing medication nonadherence [28, 29, 32, 33], and growing prescription drug spending is straining the finances of governments and patients. It is well known that inhaled drug therapy is the basic method of COPD treatment, and long-term adherence to inhaled drug therapy has been shown to improve COPD outcomes [6]. However, inhalers are more expensive than many other commonly prescribed drugs [33, 34], missing or delaying medication because of financial concerns has become increasingly common [35, 36]. In addition, the US Food and Drug Administration (FDA) banned the production and sale of all Chlorofluorocarbon (CFC) based salbutamol inhalers in 2008 to reduce environmental pollutants, but were thus replaced by more expensive hydroflurane inhalers [37]. Because exclusivity protections extend the life of branded therapies, patient drug costs are affected by prices set by manufacturers, which drives OOP costs higher for patients, especially those with private insurance [38]. This increase in cost could reduce the affordability of prescribing programs and thus reduce patient nonadherence [38]. Therefore, costs associated with COPD medication are an increasing concern, and introducing public health interventions that address barriers to this burden to reduce the incidence of CRN is imperative [29].

We also found that CRN in patients with COPD was different concerning sex. In this study, among adults with COPD who reported CRN, 68% were female, women had a nearly 50% higher risk of CRN compared to men. Sex has been identified as a factor significantly associated with CRN in a wide range of medical care [3, 39] suggesting that sex influences CRN in patients with COPD. The challenges to pay for medical care experienced by men and women may be different and need to be considered when developing interventions.

Patients with COPD often have comorbidities [40]. There were significant differences in CRN between patients with and without certain comorbidities. As reported in previous studies, we found that patients with certain comorbidities had a significantly higher risk of CRN than patients without these comorbidities [40]. Complex medication regimens, polypharmacy, the route of administration, and expensive drug cost are common and important contributors to suboptimal medication adherence among patients with certain comorbidities [12, 40]. Patients taking multiple medications, each with a different dosing pattern, are often frustrated and confused by the complex dosing regimens and the high cost of the medication, causing them to miss some doses [13]. Errors in employing inhalation techniques are common among patients using inhaled medications [3, 11, 41]. This case is unique in that if the administration technique is inaccurate, the medication will not be delivered to the lower respiratory tract of the patients, resulting in unnecessary cost waste [42]. Similarly, in some healthcare systems, the inability to pay for or reduced access to medication is associated with non-adherence. These results reinforce the importance of observing the medical and social challenges faced by patients with certain comorbidities to reduce CRN.

CRN is a major risk factor for therapeutic effects in the clinical treatment of patients. High rates of CRN warrant a broader focus on decreasing drug costs and improving health insurance coverage. Although our study provided a reference for the characteristics and risk factors of nonadherence, it has a few limitations. First, as this study was conducted in the US, our results may not be generalizable to other countries where insurance coverage, drug prices, and drug affordability differ. Second, COPD diagnosis was based on self-report, however, the rate of self-reported COPD in the NHIS dataset is consistent with the national rate of diagnosed COPD reported by the National Centre for Health Statistics [43]. Finally, due to the limitations of the database utilized in this study, several risk factors were not included the database, including disease severity in COPD patients, patient beliefs and experiences, as well as behaviors such as oral/inhaled medication usage and number of medications taken. The extent to which these risk factors affect CRN reported by patients with COPD could not be ascertained. And we did not capture the relationship between negative emotions, such as depression, and CRN in the study. However, some previous studies have found that depression was associated with medication nonadherence [3, 40]. The issue of the effect of negative emotions and CRN is an avenue for future research.

## Conclusion

In the US, one in six adults with COPD reported CRN. The influencing factors of CRN are multifaceted and necessitating more rigorous research. Health policy interventions focusing on reducing drug costs, delaying disease progression, preventing exacerbations, and reducing the risk of comorbidities may improve the economic burden of COPD and its outcomes. This report can serve as an reference for improving medication nonadherence.

### Supplementary Information

**Supplementary material 1.**

## Data Availability

Publicly available datasets were analyzed in this study. This data can be found here: https://www.cdc.gov/nchs/nhis/index.htm.

## Abbreviations

COPD: Chronic obstructive pulmonary disease
CRN: Cost-related medication nonadherence
NHIS: National health interview survey
CI: Confidence interval
OR: Odds ratio
OOP: Out-of-pocket
ACA: Affordable care act
FDA: Food and drug administration
CFC: Chlorofluorocarbon
