# Supplementary material for: Cost-related medication nonadherence in adults with COPD in the United States 2013–2020
Source: BMC Public Health. 2024 Mar 20;24:864. doi: 10.1186/s12889-024-18333-z (PMC10956194; doi:10.1186/s12889-024-18333-z)
Supplement: Supplementary file 1 — Supplementary material 1. [file 12889_2024_18333_MOESM1_ESM.docx]

# Supplemental material

## Supplemental Figure 1: Subject Selection Flowsheet


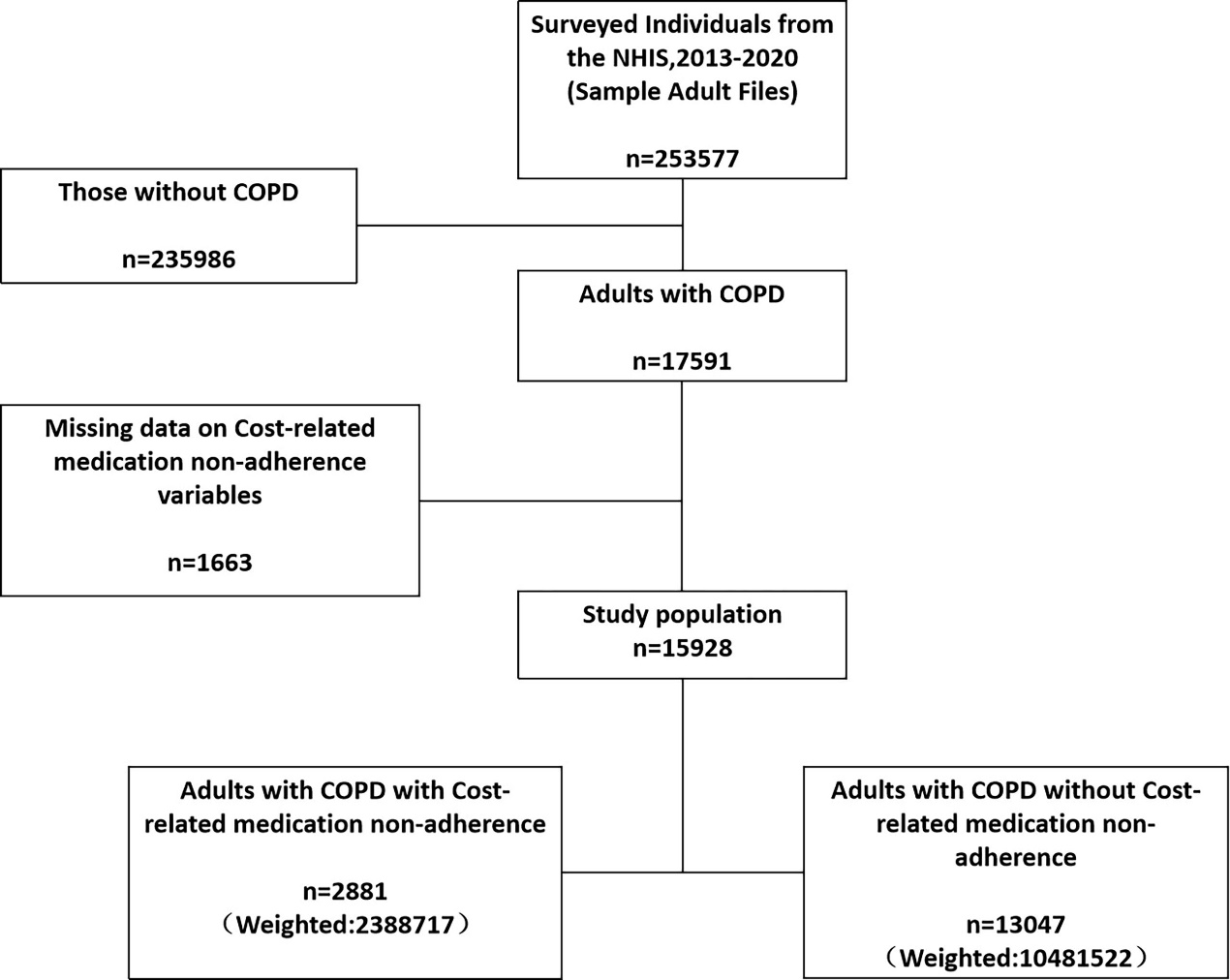


Abbreviations: COPD, chronic obstructive pulmonary disease; NHIS, National Health Interview Survey.

## Supplemental Figure 2: Temporal trends in cost-related nonadherence and its components.


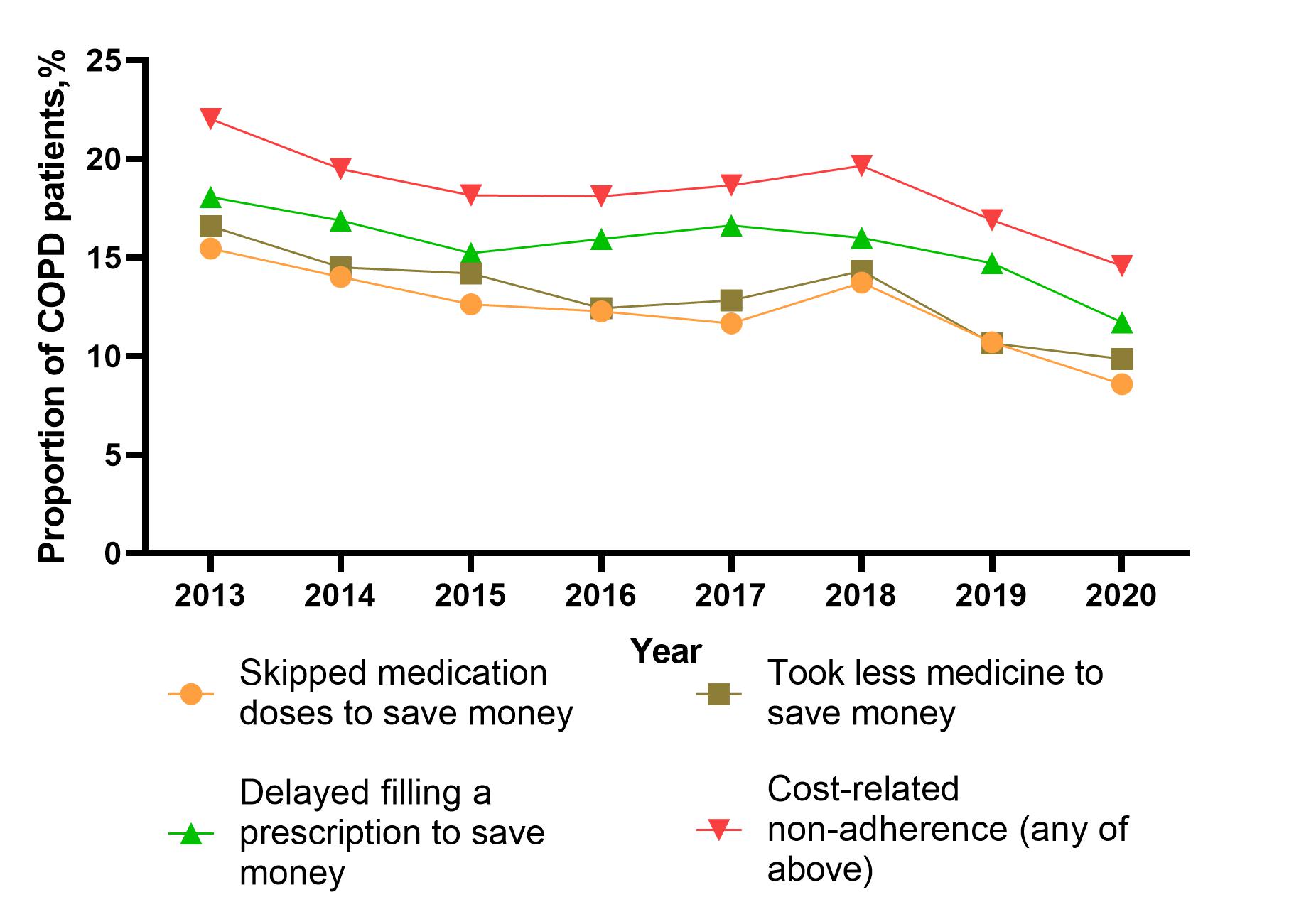


COPD indicates chronic obstructive pulmonary disease.

## Supplemental Table 1: Rates of CRN and Its Components Across Subgroups of Patients with COPD.

|  | Age <65 y | | | | Age≥65 y | | | |
| --- | --- | --- | --- | --- | --- | --- | --- | --- |
|  | (1) Skipped Medication Doses to Save Money | (2) Took Less Medicine to Save Money | (3) Delayed Filling a Prescription to Save Money | 1, 2, and/or 3=CRN | (1) Skipped Medication Doses to Save Money | (2) Took Less Medicine to Save Money | (3) Delayed Filling a Prescription to Save Money | 1, 2, and/or 3=CRN |
| Sample, n | 1,394 | 1,510 | 1,796 | 2,064 | 495 | 556 | 640 | 817 |
| Weighted sample, n (weighted %) | 1263638(16.94) | 1333011(17.87) | 1585142(21.25) | 1819370(24.39) | 345206(6.38) | 378752(7.00) | 440976(8.15) | 569211(10.52) |
| Sex |  |  |  |  |  |  |  |  |
| Male | 15.03 | 15.74 | 18.04 | 21.12 | 4.46 | 5.14 | 5.91 | 7.64 |
|  | (13.26,16.98) | (13.97,17.68) | (16.24,19.99) | (19.17,23.22) | (3.67,5.41) | (4.31,6.12) | (4.98,7.01) | (6.59,8.85) |
| Female | 18.10 | 19.16 | 23.19 | 26.37 | 7.84 | 8.41 | 9.84 | 12.70 |
|  | (16.77,19.52) | (17.79,20.62) | (21.72,24.73) | (24.84,27.96) | (6.87,8.93) | (7.37,9.59) | (8.78,11.01) | (11.44,14.07) |
| Race/ethnicity |  |  |  |  |  |  |  |  |
| Non-Hispanic white | 16.54 | 17.40 | 20.66 | 23.72 | 6.05 | 6.68 | 7.74 | 10.05 |
|  | (15.30,17.87) | (16.15,18.72) | (19.33,22.06) | (22.30,25.20) | (5.31,6.88) | (5.88,7.57) | (6.94,8.61) | (9.11,11.09) |
| Hispanic | 20.11 | 20.40 | 26.04 | 28.88 | 8.01 | 7.34 | 8.97 | 10.83 |
|  | (16.25,24.62) | (16.52,24.91) | (21.97,30.57) | (24.72,33.44) | (5.12,12.33) | (4.71,11.28) | (6.09,13.03) | (7.53,15.33) |
| Non-Hispanic black | 17.58 | 18.91 | 22.43 | 26.05 | 8.52 | 8.87 | 11.39 | 14.53 |
|  | (14.87,20.65) | (15.99,22.22) | (19.41,25.78) | (22.76,29.63) | (6.46,11.14) | (6.80,11.49) | (9.19,14.04) | (11.96,17.43) |
| Non-Hispanic Asian | 11.85 | 13.29 | 10.79 | 15.64 | 6.97 | 12.12 | 8.57 | 12.12 |
|  | (6.02,21.99) | (7.17,23.32) | (5.24,20.91) | (9.07,25.63) | (2.52,17.85) | (5.99,23.01) | (3.47,19.66) | (5.99,23.01) |
| Region |  |  |  |  |  |  |  |  |
| Northeast | 13.81 | 13.65 | 15.32 | 18.34 | 4.76 | 5.95 | 6.76 | 9.89 |
|  | (11.61,16.34) | (11.53,16.08) | (13.10,17.85) | (15.84,21.14) | (3.40,6.61) | (4.17,8.41) | (5.19,8.75) | (7.79,12.47) |
| Midwest | 17.68 | 17.81 | 21.84 | 24.92 | 7.08 | 7.76 | 8.20 | 11.47 |
|  | (15.52,20.07) | (15.59,20.27) | (19.43,24.45) | (22.31,27.73) | (5.74,8.69) | (6.33,9.47) | (6.87,9.78) | (9.72,13.49) |
| South | 18.69 | 20.39 | 23.74 | 27.30 | 6.17 | 6.67 | 8.65 | 10.37 |
|  | (16.93,20.58) | (18.59,22.33) | (21.86,25.72) | (25.34,29.36) | (5.13,7.40) | (5.65,7.87) | (7.43,10.04) | (9.05,11.86) |
| West | 14.48 | 15.64 | 19.69 | 22.03 | 7.43 | 7.70 | 8.25 | 10.19 |
|  | (12.05,17.31) | (13.13,18.52) | (17.07,22.61) | (19.30,25.02) | (5.85,9.40) | (6.09,9.70) | (6.61,10.25) | (8.34,12.39) |
| Education level |  |  |  |  |  |  |  |  |
| Less than high school | 19.13 | 21.52 | 24.27 | 27.83 | 8.10 | 8.39 | 9.85 | 12.53 |
|  | (16.64,21.89) | (19.00,24.28) | (21.52,27.26) | (24.90,30.97) | (6.52,10.01) | (6.76,10.38) | (8.07,11.98) | (10.47,14.92) |
| High school graduate | 15.77 | 17.81 | 20.94 | 24.26 | 5.29 | 6.57 | 7.41 | 9.77 |
|  | (13.64,18.16) | (15.48,20.39) | (18.58,23.51) | (21.74,26.98) | (4.22,6.60) | (5.36,8.02) | (6.11,8.96) | (8.25,11.53) |
| College or above | 16.79 | 16.89 | 20.54 | 23.47 | 6.39 | 6.81 | 8.01 | 10.30 |
|  | (15.41,18.27) | (15.56,18.32) | (19.08,22.07) | (21.94,25.07) | (5.44,7.49) | (5.82,7.95) | (7.02,9.13) | (9.13,11.60) |
| Household income |  |  |  |  |  |  |  |  |
| Low | 21.09 | 22.57 | 26.23 | 29.99 | 8.86 | 9.68 | 11.70 | 14.74 |
|  | (19.35,22.94) | (20.75,24.50) | (24.39,28.15) | (28.06,31.99) | (7.72,10.16) | (8.52,10.98) | (10.36,13.18) | (13.22,16.40) |
| Middle | 16.69 | 17.42 | 22.63 | 25.46 | 6.09 | 7.01 | 7.28 | 9.92 |
|  | (14.60,19.02) | (15.31,19.75) | (20.25,25.20) | (22.95,28.14) | (4.88,7.57) | (5.66,8.66) | (6.07,8.71) | (8.38,11.72) |
| High | 9.44 | 9.45 | 10.60 | 13.17 | 3.02 | 3.20 | 4.24 | 5.22 |
|  | (7.81,11.37) | (7.80,11.40) | (8.90,12.58) | (11.24,15.36) | (2.01,4.52) | (2.14,4.76) | (3.04,5.88) | (3.89,6.98) |
| Insurance status |  |  |  |  |  |  |  |  |
| Public | 16.91 | 18.27 | 21.48 | 25.10 | 6.97 | 7.82 | 9.37 | 11.71 |
|  | (15.32,18.64) | (16.65,20.01) | (19.74,23.33) | (23.28,27.01) | (6.09,7.97) | (6.83,8.93) | (8.32,10.54) | (10.52,13.02) |
| Private | 15.77 | 16.20 | 19.55 | 22.25 | 5.42 | 5.79 | 6.41 | 8.75 |
|  | (14.41,17.22) | (14.85,17.66) | (18.10,21.08) | (20.70,23.88) | (4.50,6.51) | (4.82,6.93) | (5.42,7.56) | (7.56,10.10) |
| Uninsured | 36.33 | 39.16 | 45.12 | 48.95 | 15.76 | 12.29 | 10.48 | 18.15 |
|  | (27.77,45.86) | (30.53,48.52) | (36.28,54.28) | (39.91,58.06) | (7.67,29.64) | (5.31,25.93) | (4.31,23.36) | (9.26,32.50) |
| Smoking status |  |  |  |  |  |  |  |  |
| Never | 13.63 | 14.37 | 17.95 | 20.61 | 6.24 | 6.87 | 8.24 | 10.78 |
|  | (12.03,15.41) | (12.79,16.11) | (16.19,19.87) | (18.72,22.63) | (4.96,7.82) | (5.44,8.63) | (6.81,9.94) | (9.04,12.80) |
| Previous | 15.17 | 15.86 | 18.41 | 22.02 | 5.62 | 6.21 | 7.24 | 9.27 |
|  | (13.29,17.26) | (13.93,18.01) | (16.29,20.73) | (19.78,24.43) | (4.80,6.57) | (5.36,7.18) | (6.30,8.31) | (8.19,10.46) |
| Current | 20.81 | 22.11 | 25.84 | 29.07 | 8.70 | 9.43 | 10.62 | 13.77 |
|  | (18.88,22.89) | (20.16,24.18) | (23.76,28.04) | (26.92,31.31) | (7.00,10.76) | (7.65,11.56) | (8.79,12.77) | (11.69,16.17) |
| Alcohol drinking status^a^ |  |  |  |  |  |  |  |  |
| Never | 15.65 | 14.93 | 17.83 | 20.94 | 7.23 | 9.38 | 9.49 | 12.10 |
|  | (12.92,18.84) | (12.39,17.89) | (15.02,21.04) | (17.90,24.33) | (5.35,9.70) | (7.24,12.06) | (7.47,11.97) | (9.77,14.89) |
| Previous | 18.58 | 20.33 | 23.42 | 26.66 | 6.43 | 7.07 | 8.17 | 10.84 |
|  | (16.25,21.17) | (17.90,23.00) | (20.87,26.17) | (23.99,29.52) | (5.31,7.76) | (5.82,8.56) | (6.92,9.61) | (9.30,12.59) |
| Current | 16.70 | 17.89 | 21.22 | 24.32 | 6.12 | 6.24 | 7.49 | 9.76 |
|  | (15.30,18.21) | (16.44,19.44) | (19.69,22.83) | (22.73,25.98) | (5.15,7.26) | (5.26,7.39) | (6.46,8.68) | (8.57,11.09) |
| Comorbidities |  |  |  |  |  |  |  |  |
| Hypertension | 19.32 | 20.08 | 22.78 | 26.17 | 6.70 | 7.06 | 8.42 | 10.80 |
|  | (17.71,21.03) | (18.44,21.83) | (21.09,24.57) | (24.38,28.05) | (5.89,7.61) | (6.25,7.96) | (7.56,9.37) | (9.82,11.87) |
| High cholesterol | 18.49 | 19.15 | 22.80 | 26.32 | 6.82 | 7.21 | 8.72 | 11.24 |
|  | (16.78,20.35) | (17.42,21.01) | (20.98,24.73) | (24.41,28.32) | (5.94,7.81) | (6.31,8.22) | (7.76,9.79) | (10.12,12.47) |
| Coronary heart disease | 20.32 | 20.56 | 24.18 | 27.34 | 7.14 | 7.58 | 8.94 | 11.63 |
|  | (17.87,23.01) | (18.23,23.12) | (21.59,26.97) | (24.63,30.23) | (6.09,8.36) | (6.54,8.76) | (7.72,10.32) | (10.29,13.11) |
| Stroke | 19.08 | 21.41 | 23.87 | 26.09 | 8.53 | 8.56 | 9.20 | 12.17 |
|  | (15.68,23.01) | (18.00,25.26) | (20.19,27.97) | (22.36,30.20) | (6.42,11.23) | (6.43,11.30) | (7.08,11.87) | (9.70,15.15) |
| Asthma | 19.29 | 20.63 | 23.86 | 27.44 | 8.59 | 9.26 | 10.98 | 14.11 |
|  | (17.74,20.94) | (18.97,22.39) | (22.14,25.67) | (25.64,29.32) | (7.31,10.07) | (8.01,10.67) | (9.63,12.49) | (12.51,15.89) |
| Cancer | 19.99 | 19.94 | 24.91 | 28.40 | 5.38 | 5.76 | 7.01 | 9.26 |
|  | (17.15,23.16) | (17.23,22.95) | (21.86,28.23) | (25.06,32.00) | (4.39,6.58) | (4.76,6.96) | (5.88,8.34) | (7.98,10.72) |
| Diabetes | 20.93 | 22.12 | 25.34 | 29.60 | 7.67 | 8.15 | 10.12 | 12.16 |
|  | (18.26,23.87) | (19.43,25.06) | (22.69,28.19) | (26.77,32.60) | (6.33,9.27) | (6.77,9.77) | (8.56,11.91) | (10.48,14.08) |
| Chronic kidney disease^a^ | 22.71 | 26.10 | 31.27 | 33.59 | 8.15 | 9.34 | 12.09 | 13.60 |
|  | (17.52,28.89) | (20.83,32.15) | (25.61,37.55) | (27.79,39.94) | (5.82,11.30) | (6.94,12.46) | (9.26,15.63) | (10.65,17.20) |
| Insufficient physical activity^a^ | 16.05 | 17.19 | 20.48 | 24.00 | 6.81 | 6.77 | 8.36 | 10.35 |
|  | (14.48,17.75) | (15.56,18.95) | (18.75,22.34) | (22.15,25.95) | (5.67,8.18) | (5.66,8.08) | (7.11,9.81) | (9.00,11.88) |
| BMI |  |  |  |  |  |  |  |  |
| Normal (<25 kg/m^2^) | 17.19 | 18.22 | 21.41 | 24.10 | 5.67 | 7.31 | 7.12 | 10.04 |
|  | (14.62,20.10) | (15.52,21.28) | (18.53,24.60) | (21.18,27.28) | (4.30,7.43) | (5.60,9.48) | (5.65,8.94) | (8.12,12.36) |
| Overweight (25-30kg/m^2^) | 14.88 | 16.36 | 19.79 | 22.48 | 5.30 | 6.03 | 6.80 | 8.78 |
|  | (13.13,16.82) | (14.50,18.42) | (17.83,21.91) | (20.36,24.76) | (4.28,6.54) | (4.96,7.32) | (5.65,8.16) | (7.48,10.29) |
| Obese(>30kg/m^2^) | 17.75 | 18.39 | 21.85 | 25.27 | 7.32 | 7.43 | 9.34 | 11.71 |
|  | (16.30,19.31) | (16.94,19.93) | (20.29,23.50) | (23.62,26.99) | (6.30,8.47) | (6.44,8.57) | (8.23,10.57) | (10.45,13.09) |

Values indicate weighted percentage (95% CI) unless otherwise indicated. Abbreviations: COPD, chronic obstructive pulmonary disease; CRN, cost-related medication nonadherence; BMI, body mass index.

Missing values of covariates less than 5% were filled with multiple imputations.

a Data on alcohol drinking status, chronic kidney disease, and insufficient physical activity were not surveyed in 2019 which were excluded in the corresponding analysis

## Supplemental Table 2: National estimates for rates of CRN among adults with COPD overall, and among major patient subgroups.

|  | (1) Skipped Medication Doses to Save Money | (2) Took Less Medicine to Save Money | (3) Delayed Filling a Prescription to Save Money | 1, 2, and/or 3=CRN |
| --- | --- | --- | --- | --- |
| Sample, n | 1,889 | 2,066 | 2,436 | 2,881 |
| Weighted sample, n (weighted %) | 1608780(12.50) | 1711742(13.3) | 2025776(15.74) | 2388716(18.56) |
|  | Weighted % (95% CI) | Weighted % (95% CI) | Weighted % (95% CI) | Weighted % (95% CI) |
| Age category, y |  |  |  |  |
| 18–64 | 16.94(15.85,18.10) | 17.87(16.76,19.05) | 21.25(20.07,22.48) | 24.39(23.14,25.69) |
| ≥65 | 6.38(5.70,7.13) | 7.00(6.28,7.80) | 8.15(7.41,8.95) | 10.52(9.64,11.46) |
| Sex |  |  |  |  |
| Male | 10.23(9.15,11.42) | 10.93(9.86,12.10) | 12.54(11.42,13.75) | 15.01(13.78,16.32) |
| Female | 14.02(13.10,14.98) | 14.88(13.94,15.88) | 17.88(16.87,18.93) | 20.93(19.86,22.03) |
| Race/ethnicity |  |  |  |  |
| Non-Hispanic white | 11.85(11.07,12.68) | 12.61(11.81,13.45) | 14.89(14.03,15.79) | 17.61(16.69,18.58) |
| Hispanic | 16.30(13.26,19.88) | 16.29(13.41,19.66) | 20.67(17.51,24.24) | 23.21(19.97,26.79) |
| Non-Hispanic black | 14.67(12.66,16.94) | 15.69(13.51,18.14) | 18.89(16.66,21.35) | 22.35(19.90,25.02) |
| Non-Hispanic Asian | 11.94(8.51,16.50) | 14.74(11.03,19.41) | 14.10(10.52,18.64) | 17.64(13.73,22.37) |
| Region |  |  |  |  |
| Northeast | 9.82(8.49,11.32) | 10.25(8.88,11.81) | 11.55(10.10,13.17) | 14.62(13.00,16.40) |
| Midwest | 13.35(11.92,14.93) | 13.71(12.24,15.32) | 16.28(14.73,17.95) | 19.43(17.68,21.32) |
| South | 13.53(12.39,14.77) | 14.75(13.58,16.00) | 17.53(16.24,18.89) | 20.33(19.01,21.72) |
| West | 11.38(9.81,13.17) | 12.15(10.53,13.98) | 14.66(12.98,16.52) | 16.82(15.05,18.76) |
| Education level |  |  |  |  |
| Less than high school | 14.33(12.73,16.11) | 15.82(14.18,17.61) | 18.01(16.16,20.02) | 21.18(19.22,23.28) |
| High school graduate | 10.98(9.70,12.41) | 12.68(11.28,14.21) | 14.76(13.31,16.34) | 17.65(16.09,19.31) |
| College or above | 12.62(11.69,13.60) | 12.85(11.94,13.80) | 15.51(14.55,16.52) | 18.18(17.16,19.26) |
| Household income |  |  |  |  |
| Low | 16.67(15.47,17.94) | 17.91(16.66,19.23) | 20.97(19.69,22.32) | 24.47(23.12,25.88) |
| Middle | 11.47(10.25,12.82) | 12.30(11.01,13.71) | 15.07(13.72,16.53) | 17.81(16.33,19.40) |
| High | 6.78(5.71,8.03) | 6.85(5.77,8.12) | 7.96(6.84,9.24) | 9.87(8.61,11.28) |
| Insurance status |  |  |  |  |
| Public | 12.09(11.13,13.12) | 13.20(12.22,14.24) | 15.61(14.52,16.76) | 18.60(17.46,19.80) |
| Private | 11.99(11.07,12.97) | 12.40(11.47,13.39) | 14.75(13.75,15.81) | 17.32(16.24,18.45) |
| Uninsured | 33.83(26.09,42.54) | 35.89(28.12,44.47) | 40.90(32.89,49.44) | 45.20(37.03,53.64) |
| Smoking status |  |  |  |  |
| Never | 10.94(9.78,12.21) | 11.64(10.49,12.89) | 14.41(13.15,15.78) | 17.03(15.67,18.48) |
| Previous | 9.46(8.53,10.48) | 10.09(9.14,11.12) | 11.73(10.68,12.87) | 14.39(13.25,15.62) |
| Current | 17.68(16.14,19.33) | 18.83(17.27,20.49) | 21.90(20.24,23.66) | 25.11(23.40,26.90) |
| Alcohol drinking status^a^ |  |  |  |  |
| Never | 11.70(10.00,13.66) | 12.33(10.63,14.25) | 13.92(12.13,15.93) | 16.79(14.83,18.96) |
| Previous | 12.53(11.16,14.03) | 13.72(12.31,15.27) | 15.82(14.35,17.41) | 18.78(17.17,20.50) |
| Current | 13.01(12.01,14.08) | 13.83(12.79,14.93) | 16.43(15.34,17.58) | 19.24(18.10,20.44) |
| Comorbidities |  |  |  |  |
| Hypertension | 12.97(12.05,13.95) | 13.53(12.61,14.51) | 15.56(14.57,16.61) | 18.45(17.39,19.55) |
| High cholesterol | 12.71(11.73,13.75) | 13.23(12.24,14.28) | 15.82(14.78,16.92) | 18.84(17.72,20.03) |
| Coronary heart disease | 13.12(11.80,14.56) | 13.47(12.22,14.82) | 15.85(14.41,17.40) | 18.75(17.24,20.37) |
| Stroke | 13.16(11.15,15.48) | 14.21(12.22,16.45) | 15.65(13.53,18.03) | 18.29(16.05,20.76) |
| Asthma | 15.68(14.55,16.89) | 16.79(15.60,18.06) | 19.51(18.29,20.80) | 22.95(21.63,24.32) |
| Cancer | 10.84(9.54,12.30) | 11.06(9.84,12.41) | 13.70(12.30,15.24) | 16.42(14.87,18.10) |
| Diabetes | 14.51(12.91,16.28) | 15.35(13.77,17.08) | 17.97(16.33,19.74) | 21.16(19.40,23.03) |
| Chronic kidney disease^a^ | 14.90(12.03,18.31) | 17.11(14.23,20.43) | 20.98(17.71,24.67) | 22.86(19.53,26.58) |
| Insufficient physical activity^a^ | 12.63(11.53,13.81) | 13.33(12.21,14.54) | 15.99(14.77,17.29) | 18.94(17.65,20.30) |
| BMI |  |  |  |  |
| Normal (<25kg/㎡) | 11.74(10.13,13.57) | 13.06(11.35,14.99) | 14.65(12.86,16.65) | 17.45(15.58,19.49) |
| Overweight (25-30kg/㎡) | 10.44(9.40,11.57) | 11.57(10.46,12.78) | 13.76(12.58,15.04) | 16.13(14.83,17.51) |
| Obese(>30kg/㎡) | 13.80(12.78,14.89) | 14.24(13.24,15.30) | 17.11(16.03,18.26) | 20.13(18.98,21.34) |

Abbreviations: COPD, chronic obstructive pulmonary disease; CRN, cost-related medication nonadherence; BMI, body mass index.

Missing values of covariates less than 5% were filled with multiple imputations.

^a^ Data on alcohol drinking status, chronic kidney disease, and insufficient physical activity were not surveyed in 2019 which were excluded in the corresponding analysis.

## Supplemental Table 3: Predictors of CRN among adults with COPD, above and below 65 years of age.

|  | OR (95% CI) | |
| --- | --- | --- |
| Variable | Age <65 years | Age ≥65 years |
| Sex |  |  |
| Male | Reference | Reference |
| Female | 1.34(1.16,1.54) | 1.76(1.44,2.14) |
| Race/ethnicity |  |  |
| Non-Hispanic white | Reference | Reference |
| Hispanic | 1.31(1.04,1.64) | 1.08(0.72,1.64) |
| Non-Hispanic black | 1.13(0.93,1.37) | 1.52(1.18,1.95) |
| Non-Hispanic Asian | 0.87(0.60,1.26) | 1.14(0.64,2.03) |
| Region |  |  |
| Northeast | Reference | Reference |
| Midwest | 1.47(1.17,1.85) | 1.18(0.86,1.63) |
| South | 1.67(1.36,2.05) | 1.05(0.78,1.43) |
| West | 1.25(0.98,1.60) | 1.03(0.73,1.46) |
| Education level |  |  |
| College or above | Reference | Reference |
| High school graduate | 1.04(0.88,1.24) | 0.94(0.74,1.20) |
| Less than high school | 1.26(1.06,1.49) | 1.24(0.98,1.59) |
| Household income |  |  |
| High | Reference | Reference |
| Middle | 2.15(1.72,2.69) | 1.77(1.26,2.49) |
| Low | 2.71(2.23,3.30) | 2.92(2.13,4.02) |
| Insurance status |  |  |
| Public | Reference | Reference |
| Private | 0.85(0.75,0.98) | 0.72(0.60,0.87) |
| Uninsured | 2.86(1.97,4.15) | 1.67(0.76,3.65) |
| Smoking status |  |  |
| Never | Reference | Reference |
| Previous | 1.08(0.91,1.31) | 0.85(0.67,1.07) |
| Current | 1.58(1.34,1.86) | 1.32(1.01,1.73) |
| Alcohol drinking status^a^ |  |  |
| Never | Reference | Reference |
| Previous | 1.37(1.08,1.75) | 0.88(0.66,1.19) |
| Current | 1.21(0.98,1.50) | 0.79(0.59,1.04) |
| Comorbidities |  |  |
| Hypertension | 1.23(1.07,1.40) | 1.12(0.91,1.39) |
| High cholesterol | 1.21(1.06,1.38) | 1.22(1.00,1.50) |
| Coronary heart disease | 1.24(1.05,1.46) | 1.23(1.02,1.48) |
| Stroke | 1.10(0.90,1.36) | 1.21(0.93,1.59) |
| Asthma | 1.34(1.19,1.52) | 1.68(1.39,2.04) |
| Cancer | 1.27(1.05,1.55) | 0.82(0.67,1.00) |
| Diabetes | 1.40(1.21,1.63) | 1.26(1.02,1.54) |
| Chronic kidney disease^a^ | 1.61(1.22,2.13) | 1.36(1.01,1.84) |
| Insufficient physical activity^a^ | 1.17(0.92,1.49) | 1.63(1.06,2.52) |
| BMI |  |  |
| Normal(<25kg/㎡) | Reference | Reference |
| Overweight (25-30kg/㎡) | 0.91(0.74,1.12) | 0.86(0.65,1.15) |
| Obese(>30kg/㎡) | 1.06(0.89,1.30) | 1.19(0.91,1.55) |

Abbreviations: COPD, chronic obstructive pulmonary disease; CI, confidence interval; BMI, body mass index; OR, Odds ratio; CRN, cost-related medication nonadherence.

Missing values of covariates less than 5% were filled with multiple imputations.

a Data on alcohol drinking status, chronic kidney disease, and insufficient physical activity were not surveyed in 2019 which were excluded in the corresponding analysis.

## Supplemental Table 4: Characteristics Among Adults with Emphysema^a^ Based on Whether They Reported Cost-Related Nonadherence.

| Variable | No Cost-Related Nonadherence, Weighted % (95% CI) | Cost-Related Nonadherence, Weighted % (95% CI) | *P* |
| --- | --- | --- | --- |
| Sample, n | 2,634 | 588 |  |
| Weighted sample, n (weighted %) | 1988810(81.20) | 460463(18.80) |  |
| Age category, y |  |  | <0.001 |
| 18–64 | 40.46(38.05,42.93) | 65.55(60.67,70.12) |  |
| ≥65 | 59.54(57.07,61.95) | 34.45(29.88,39.33) |  |
| Female | 45.64(42.97,48.33) | 63.56(58.22,68.58) | <0.001 |
| Race/ethnicity |  |  | 0.454 |
| Non-Hispanic white | 83.99(81.98,85.82) | 80.85(76.30,84.70) |  |
| Hispanic | 5.93(4.62,7.57) | 5.27(3.56,7.76) |  |
| Non-Hispanic black | 7.42(6.24,8.80) | 9.61(6.90,13.22) |  |
| Non-Hispanic Asian | 1.67(1.19,2.32) | 2.57(1.10,5.88) |  |
| Region |  |  | 0.064 |
| Northeast | 17.34(15.25,19.65) | 15.82(11.81,20.88) |  |
| Midwest | 26.24(23.45,29.24) | 25.88(21.11,31.30) |  |
| South | 39.13(36.09,42.26) | 45.88(40.40,51.46) |  |
| West | 17.29(15.18,19.61) | 12.41(9.36,16.29) |  |
| Education level |  |  | 0.069 |
| Less than high school | 21.46(19.56,23.49) | 25.72(21.54,30.38) |  |
| High school graduate | 25.99(23.74,28.37) | 28.16(24.10,32.60) |  |
| College or above | 52.55(49.95,55.14) | 46.13(41.10,51.24) |  |
| Household income |  |  | <0.001 |
| Low | 49.24(46.62,51.87) | 69.29(64.32,73.86) |  |
| Middle | 29.31(26.98,31.75) | 24.03(19.88,28.74) |  |
| High | 21.45(19.18,23.91) | 6.68(4.58,9.64) |  |
| Insurance status |  |  | 0.055 |
| Public | 61.03(58.43,63.57) | 58.25(53.18,63.15) |  |
| Private | 38.09(35.56,40.69) | 38.27(33.13,43.70) |  |
| Uninsured | 0.88(0.52,1.47) | 3.48(1.71,6.95) |  |
| Smoking status |  |  | <0.001 |
| Never | 11.32(9.81,13.02) | 13.07(9.84,17.16) |  |
| Previous | 53.13(50.50,55.75) | 35.23(30.47,40.31) |  |
| Current | 35.55(33.17,38.00) | 51.70(46.53,56.84) |  |
| Alcohol drinking status^b^ |  |  | 0.429 |
| Never | 13.69(12.18,15.35) | 16.33(12.83,20.56) |  |
| Previous | 37.25(34.90,39.67) | 35.54(30.70,40.69) |  |
| Current | 49.06(46.55,51.58) | 48.13(42.61,53.70) |  |
| Comorbidities |  |  |  |
| Hypertension | 67.27(64.79,69.67) | 69.36(64.19,74.08) | 0.448 |
| High cholesterol | 56.96(54.45,59.42) | 60.77(56.00,65.34) | 0.171 |
| Coronary heart disease | 47.05(44.42,49.69) | 49.64(44.23,55.07) | 0.412 |
| Stroke | 16.14(14.35,18.12) | 14.11(11.00,17.91) | 0.312 |
| Asthma | 38.04(35.60,40.54) | 49.11(43.87,54.37) | 0.000 |
| Cancer | 27.20(25.07,29.44) | 22.43(18.26,27.25) | 0.069 |
| Diabetes | 24.36(22.17,26.70) | 27.11(23.05,31.60) | 0.269 |
| Chronic kidney disease^b^ | 9.89(8.56,11.41) | 14.01(10.66,18.19) | 0.043 |
| Insufficient physical activity^b^ | 88.14(85.40,90.43) | 87.40(80.49,92.10) | 0.815 |
| BMI |  |  | 0.248 |
| Normal(<25kg/㎡) | 28.53(26.27,30.89) | 28.27(23.91,33.09) |  |
| Overweight(25-30kg/㎡) | 31.28(29.00,33.65) | 27.48(23.41,31.97) |  |
| Obese(>30kg/㎡) | 40.19(37.69,42.75) | 44.24(39.48,49.11) |  |

BMI indicates body mass index.

a Data on emphysema were not surveyed from 2019 to 2020.

Missing values of covariates less than 5% were filled with multiple imputations.

a Data on alcohol drinking status, chronic kidney disease, and insufficient physical activity were not surveyed in 2019 which were excluded in the corresponding analysis.

## Supplemental Table 5: Rates of CRN and Its Components Across Subgroups of Patients with Emphysema^a^.

|  | Age <65 y | | | | Age≥65 y | | | |
| --- | --- | --- | --- | --- | --- | --- | --- | --- |
|  | (1) Skipped Medication Doses to Save Money | (2) Took Less Medicine to Save Money | (3) Delayed Filling a Prescription to Save Money | 1, 2, and/or 3=CRN | (1) Skipped Medication Doses to Save Money | (2) Took Less Medicine to Save Money | (3) Delayed Filling a Prescription to Save Money | 1, 2, and/or 3=CRN |
| Sample, n | 255 | 277 | 311 | 356 | 138 | 161 | 187 | 232 |
| Weighted sample, n (weighted %) | 221648(20.03) | 237915(21.50) | 267240(24.15) | 301765(27.27) | 94660(7.05) | 107415(8.00) | 124199(9.25) | 158572(11.91) |
| Sex |  |  |  |  |  |  |  |  |
| Male | 13.62 | 15.71 | 17.58 | 20.51 | 4.32 | 6.00 | 6.15 | 7.86 |
|  | (10.26,17.85) | (11.99,20.32) | (13.88,22.02) | (16.51,25.18) | (3.08,6.04) | (4.47,8.02) | (4.61,8.16) | (6.09,10.10) |
| Female | 26.38 | 27.22 | 30.65 | 33.97 | 10.00 | 10.18 | 12.60 | 16.10 |
|  | (22.22,31.01) | (22.99,31.90) | (26.30,35.38) | (29.47,38.77) | (7.64,12.99) | (7.89,13.03) | (10.02,15.74) | (13.25,19.41) |
| Race/ethnicity |  |  |  |  |  |  |  |  |
| Non-Hispanic white | 19.51 | 20.98 | 24.10 | 27.05 | 6.81 | 7.59 | 8.76 | 11.11 |
|  | (16.71,22.66) | (18.02,24.27) | (21.00,27.51) | (23.79,30.58) | (5.32,8.67) | (6.07,9.45) | (7.08,10.78) | (9.25,13.29) |
| Hispanic | 20.85 | 21.72 | 21.78 | 24.75 | 7.58 | 7.58 | 9.89 | 11.05 |
|  | (11.62,34.56) | (12.20,35.64) | (12.27,35.67) | (14.51,38.91) | (3.63,15.15) | (3.63,15.15) | (4.90,18.94) | (5.69,20.39) |
| Non-Hispanic black | 20.29 | 22.55 | 22.61 | 26.93 | 6.74 | 9.13 | 11.80 | 18.65 |
|  | (11.36,33.59) | (13.37,35.45) | (13.42,35.51) | (17.30,39.36) | (3.44,12.78) | (5.12,15.78) | (7.34,18.43) | (12.54,26.81) |
| Non-Hispanic Asian | 43.70 | 31.43 | 31.69 | 43.95 | 15.40 | 20.91 | 18.68 | 20.91 |
|  | (16.10,75.84) | (7.68,71.64) | (7.85,71.65) | (16.32,75.93) | (4.24,42.82) | (7.47,46.39) | (5.89,45.73) | (7.47,46.39) |
| Region |  |  |  |  |  |  |  |  |
| Northeast | 21.03 | 21.23 | 24.89 | 27.38 | 5.66 | 6.73 | 7.62 | 9.84 |
|  | (14.45,29.57) | (14.35,30.25) | (17.65,33.87) | (19.82,36.53) | (2.97,10.52) | (3.59,12.25) | (4.44,12.79) | (6.28,15.11) |
| Midwest | 18.60 | 20.46 | 23.92 | 26.70 | 5.78 | 7.36 | 7.55 | 10.67 |
|  | (13.62,24.88) | (14.94,27.36) | (18.23,30.72) | (20.81,33.55) | (3.73,8.84) | (4.89,10.95) | (4.96,11.33) | (7.56,14.85) |
| South | 22.57 | 24.31 | 26.09 | 30.23 | 8.06 | 9.47 | 11.49 | 13.80 |
|  | (18.49,27.25) | (20.07,29.12) | (21.77,30.93) | (25.65,35.23) | (5.64,11.38) | (7.11,12.52) | (8.76,14.95) | (10.82,17.44) |
| West | 14.34 | 15.67 | 18.05 | 19.62 | 7.87 | 6.90 | 8.16 | 10.89 |
|  | (8.85,22.38) | (10.04,23.62) | (12.66,25.08) | (13.49,27.66) | (5.25,11.62) | (4.41,10.63) | (5.44,12.07) | (7.68,15.22) |
| Education level |  |  |  |  |  |  |  |  |
| Less than high school | 20.86 | 25.15 | 25.36 | 29.92 | 7.99 | 9.45 | 12.58 | 14.00 |
|  | (15.72,27.14) | (19.37,31.97) | (19.66,32.06) | (23.77,36.88) | (5.39,11.70) | (6.44,13.67) | (8.98,17.37) | (10.20,18.91) |
| High school graduate | 22.19 | 24.20 | 26.57 | 28.84 | 6.33 | 8.80 | 9.56 | 12.48 |
|  | (17.03,28.38) | (18.83,30.52) | (20.98,33.02) | (23.12,35.33) | (4.00,9.87) | (5.99,12.75) | (6.62,13.60) | (9.21,16.69) |
| College or above | 18.51 | 18.29 | 22.21 | 25.11 | 7.05 | 7.08 | 7.82 | 10.67 |
|  | (15.14,22.43) | (14.95,22.19) | (18.50,26.43) | (21.27,29.39) | (5.12,9.64) | (5.28,9.44) | (5.89,10.31) | (8.39,13.48) |
| Household income |  |  |  |  |  |  |  |  |
| Low | 23.48 | 26.02 | 27.31 | 31.57 | 10.85 | 11.80 | 15.19 | 17.91 |
|  | (19.86,27.54) | (22.20,30.24) | (23.54,31.44) | (27.52,35.92) | (8.53,13.71) | (9.43,14.68) | (12.34,18.57) | (14.87,21.42) |
| Middle | 20.13 | 21.02 | 26.98 | 28.47 | 5.95 | 7.03 | 7.07 | 10.09 |
|  | (14.51,27.24) | (15.09,28.49) | (20.39,34.77) | (21.76,36.28) | (3.67,9.51) | (4.57,10.68) | (4.71,10.49) | (7.25,13.88) |
| High | 8.40 | 6.92 | 8.94 | 10.93 | 2.29 | 4.06 | 2.72 | 4.51 |
|  | (4.84,14.19) | (3.77,12.36) | (5.29,14.71) | (6.85,16.99) | (0.80,6.34) | (1.95,8.28) | (1.02,7.05) | (2.30,8.65) |
| Insurance status |  |  |  |  |  |  |  |  |
| Public | 17.26 | 18.40 | 21.59 | 24.33 | 7.61 | 8.77 | 10.32 | 12.99 |
|  | (14.30,20.67) | (15.31,21.95) | (18.27,25.33) | (20.83,28.21) | (5.84,9.88) | (6.85,11.15) | (8.29,12.78) | (10.70,15.69) |
| Private | 22.69 | 24.47 | 26.31 | 30.23 | 5.94 | 6.61 | 7.56 | 9.84 |
|  | (18.20,27.90) | (19.69,29.99) | (21.45,31.83) | (25.11,35.90) | (4.13,8.47) | (4.63,9.36) | (5.41,10.47) | (7.42,12.94) |
| Uninsured | 50.83 | 55.68 | 57.44 | 57.44 | 21.41 | 21.41 | 10.92 | 21.41 |
|  | (25.52,75.72) | (29.89,78.74) | (31.45,79.88) | (31.45,79.88) | (5.32,56.91) | (5.32,56.91) | (1.50,49.63) | (5.32,56.91) |
| Smoking status |  |  |  |  |  |  |  |  |
| Never | 24.29 | 25.90 | 24.32 | 30.82 | 9.10 | 10.38 | 13.38 | 14.71 |
|  | (15.82,35.39) | (17.22,37.00) | (16.32,34.62) | (21.69,41.75) | (5.05,15.83) | (6.02,17.32) | (8.43,20.56) | (9.58,21.91) |
| Previous | 15.36 | 16.16 | 19.66 | 21.88 | 5.65 | 6.10 | 7.29 | 9.52 |
|  | (11.44,20.31) | (12.17,21.15) | (15.18,25.07) | (17.31,27.25) | (4.15,7.65) | (4.61,8.03) | (5.54,9.53) | (7.56,11.93) |
| Current | 22.12 | 23.95 | 26.88 | 29.93 | 9.59 | 11.69 | 12.14 | 16.22 |
|  | (18.51,26.20) | (20.18,28.19) | (23.05,31.10) | (25.89,34.31) | (6.78,13.38) | (8.57,15.76) | (9.20,15.86) | (12.69,20.51) |
| Alcohol drinking status^b^ |  |  |  |  |  |  |  |  |
| Never | 22.36 | 23.09 | 22.40 | 28.09 | 9.85 | 13.71 | 14.52 | 17.93 |
|  | (15.30,31.47) | (15.69,32.63) | (15.30,31.57) | (20.08,37.79) | (5.64,16.65) | (8.81,20.74) | (9.63,21.30) | (12.46,25.12) |
| Previous | 20.23 | 21.50 | 25.70 | 28.60 | 5.69 | 6.56 | 7.83 | 10.00 |
|  | (15.83,25.48) | (16.99,26.84) | (20.89,31.19) | (23.63,34.14) | (3.98,8.08) | (4.75,8.99) | (5.74,10.60) | (7.65,12.97) |
| Current | 19.35 | 21.11 | 23.73 | 26.24 | 7.10 | 7.12 | 8.44 | 11.12 |
|  | (15.90,23.34) | (17.34,25.45) | (19.96,27.96) | (22.25,30.67) | (5.26,9.51) | (5.36,9.39) | (6.42,11.03) | (8.84,13.89) |
| Comorbidities |  |  |  |  |  |  |  |  |
| Hypertension | 22.12 | 23.91 | 25.49 | 29.49 | 7.20 | 8.24 | 9.68 | 12.34 |
|  | (18.51,26.20) | (20.16,28.11) | (21.68,29.73) | (25.52,33.80) | (5.60,9.22) | (6.58,10.28) | (7.85,11.87) | (10.31,14.70) |
| High cholesterol | 20.31 | 22.75 | 26.99 | 29.60 | 7.68 | 8.53 | 10.24 | 13.01 |
|  | (16.90,24.22) | (19.02,26.98) | (22.93,31.47) | (25.42,34.15) | (5.87,9.98) | (6.66,10.86) | (8.13,12.81) | (10.69,15.75) |
| Coronary heart disease | 22.29 | 24.05 | 26.74 | 29.39 | 7.94 | 9.33 | 10.68 | 13.15 |
|  | (17.67,27.71) | (19.27,29.58) | (21.80,32.35) | (24.20,35.18) | (5.82,10.73) | (7.11,12.14) | (8.28,13.68) | (10.50,16.34) |
| Stroke | 12.15 | 15.56 | 18.83 | 20.03 | 9.84 | 9.40 | 10.44 | 14.47 |
|  | (7.86,18.31) | (10.66,22.16) | (13.41,25.78) | (14.48,27.05) | (5.83,16.12) | (5.53,15.55) | (6.60,16.14) | (9.76,20.93) |
| Asthma | 22.32 | 24.76 | 26.60 | 29.87 | 9.24 | 10.54 | 13.18 | 14.93 |
|  | (18.43,26.75) | (20.60,29.45) | (22.36,31.32) | (25.44,34.70) | (6.87,12.31) | (8.03,13.72) | (10.38,16.60) | (11.96,18.49) |
| Cancer | 20.88 | 20.12 | 26.52 | 30.15 | 5.36 | 5.99 | 7.86 | 9.82 |
|  | (14.94,28.41) | (14.28,27.59) | (19.74,34.62) | (23.05,38.36) | (3.49,8.15) | (4.09,8.67) | (5.55,11.03) | (7.21,13.25) |
| Diabetes | 21.52 | 24.89 | 27.76 | 33.10 | 6.84 | 8.45 | 9.82 | 11.63 |
|  | (16.29,27.87) | (19.26,31.52) | (21.72,34.74) | (26.83,40.04) | (4.51,10.26) | (5.80,12.15) | (6.93,13.72) | (8.44,15.83) |
| Chronic kidney disease^b^ | 26.19 | 32.27 | 34.47 | 37.96 | 11.25 | 13.04 | 16.04 | 16.36 |
|  | (16.94,38.16) | (22.13,44.39) | (24.26,46.33) | (27.23,50.02) | (6.72,18.25) | (8.06,20.41) | (10.59,23.56) | (10.88,23.85) |
| Insufficient physical activity^b^ | 18.84 | 18.96 | 21.91 | 26.10 | 5.73 | 6.22 | 7.81 | 9.19 |
|  | (14.67,23.85) | (14.75,24.04) | (17.33,27.31) | (21.24,31.63) | (4.02,8.10) | (4.43,8.69) | (5.75,10.53) | (6.95,12.06) |
| BMI |  |  |  |  |  |  |  |  |
| Normal(<25kg/㎡) | 22.95 | 23.33 | 25.03 | 28.79 | 6.70 | 7.70 | 8.20 | 11.23 |
|  | (17.94,28.85) | (18.21,29.37) | (20.00,30.83) | (23.24,35.06) | (4.47,9.93) | (5.35,10.97) | (5.84,11.40) | (8.42,14.84) |
| Overweight (25-30kg/㎡) | 17.27 | 18.29 | 20.28 | 24.32 | 6.68 | 8.81 | 9.63 | 11.59 |
|  | (13.12,22.40) | (13.87,23.73) | (15.79,25.65) | (19.34,30.10) | (4.39,10.03) | (6.19,12.40) | (6.94,13.22) | (8.64,15.39) |
| Obese(>30kg/㎡) | 20.04 | 22.42 | 26.06 | 28.22 | 7.64 | 7.55 | 9.74 | 12.46 |
|  | (16.19,24.53) | (18.41,27.01) | (21.77,30.86) | (23.83,33.08) | (5.53,10.48) | (5.62,10.08) | (7.33,12.85) | (9.77,15.77) |

Values indicate weighted percentage (95% CI), unless otherwise indicated. Abbreviations: CRN, cost-related medication nonadherence; BMI, body mass index.

a Data on emphysema were not surveyed from 2019 to 2020.

Missing values of covariates less than 5% were filled with multiple imputations.

b Data on alcohol drinking status, chronic kidney disease, and insufficient physical activity were not surveyed in 2019 which were excluded in the corresponding analysis.

## Supplemental Table 6: Characteristics Among Adults with Chronic bronchitis^a^ Based on Whether They Reported Cost-Related Nonadherence.

| Variable | No Cost-Related Nonadherence, Weighted % (95% CI) | Cost-Related Nonadherence, Weighted % (95% CI) | P Value |
| --- | --- | --- | --- |
| Sample, n | 5,571 | 1,618 |  |
| Weighted sample, n (weighted %) | 4641500(77.74) | 1329043(22.26) |  |
| Age category, y |  |  | <0.001 |
| 18–64 | 67.23(65.55,68.85) | 81.54(78.99,83.84) |  |
| ≥65 | 32.77(31.15,34.45) | 18.46(16.16,21.01) |  |
| Female | 67.98(66.28,69.63) | 72.50(69.25,75.53) | 0.011 |
| Race/ethnicity |  |  | 0.001 |
| Non-Hispanic white | 75.93(74.29,77.50) | 70.56(67.43,73.51) |  |
| Hispanic | 8.35(7.34,9.48) | 10.82(8.82,13.20) |  |
| Non-Hispanic black | 12.23(11.03,13.53) | 15.97(13.68,18.57) |  |
| Non-Hispanic Asian | 2.31(1.82,2.93) | 1.49(0.86,2.58) |  |
| Region |  |  | 0.001 |
| Northeast | 17.72(15.90,19.70) | 12.48(10.51,14.75) |  |
| Midwest | 22.88(20.84,25.05) | 25.13(21.73,28.87) |  |
| South | 42.25(39.98,44.56) | 46.07(42.47,49.71) |  |
| West | 17.15(15.56,18.87) | 16.32(13.85,19.15) |  |
| Education level |  |  | 0.000 |
| Less than high school | 12.07(11.09,13.13) | 16.86(14.73,19.23) |  |
| High school graduate | 20.23(18.82,21.71) | 20.38(18.00,22.99) |  |
| College or above | 67.70(66.00,69.35) | 62.75(59.58,65.82) |  |
| Household income |  |  | <0.001 |
| Low | 39.44(37.59,41.31) | 58.57(55.37,61.70) |  |
| Middle | 28.85(27.16,30.60) | 27.53(24.73,30.52) |  |
| High | 31.71(29.94,33.55) | 13.89(11.61,16.54) |  |
| Insurance status |  |  | 0.003 |
| Public | 43.88(42.07,45.69) | 47.14(44.02,50.28) |  |
| Private | 55.02(53.19,56.85) | 49.45(46.29,52.62) |  |
| Uninsured | 1.10(0.75,1.62) | 3.41(2.13,5.42) |  |
| Smoking status |  |  | <0.001 |
| Never | 42.94(41.17,44.73) | 35.05(31.98,38.25) |  |
| Previous | 31.15(29.56,32.77) | 25.58(22.90,28.44) |  |
| Current | 25.92(24.37,27.52) | 39.37(36.06,42.78) |  |
| Alcohol drinking status^b^ |  |  | 0.024 |
| Never | 18.98(17.56,20.50) | 15.49(13.31,17.96) |  |
| Previous | 23.20(21.71,24.76) | 26.29(23.43,29.37) |  |
| Current | 57.82(56.01,59.61) | 58.22(54.76,61.60) |  |
| Comorbidities |  |  |  |
| Hypertension | 54.09(52.28,55.88) | 57.23(53.87,60.53) | 0.099 |
| High cholesterol | 46.14(44.41,47.89) | 49.25(45.81,52.69) | 0.101 |
| Coronary heart disease | 29.24(27.65,30.88) | 33.16(29.92,36.57) | 0.045 |
| Stroke | 8.45(7.54,9.47) | 9.02(7.51,10.79) | 0.549 |
| Asthma | 43.16(41.45,44.88) | 53.14(49.65,56.60) | <0.001 |
| Cancer | 17.46(16.20,18.78) | 16.41(14.27,18.81) | 0.443 |
| Diabetes | 19.59(18.25,21.00) | 24.76(22.09,27.65) | 0.001 |
| Chronic kidney disease^b^ | 6.82(6.06,7.67) | 9.33(7.41,11.68) | 0.036 |
| Insufficient physical activity^b^ | 80.17(78.11,82.07) | 82.75(78.95,85.99) | 0.186 |
| BMI |  |  | 0.034 |
| Normal(<25kg/㎡) | 20.08(18.73,21.51) | 17.17(14.59,20.10) |  |
| Overweight (25-30kg/㎡) | 27.66(26.08,29.31) | 25.89(23.18,28.80) |  |
| Obese(>30kg/㎡) | 52.25(50.45,54.05) | 56.94(53.66,60.15) |  |

BMI indicates body mass index.

a Data on Chronic bronchitis were not surveyed from 2019 to 2020.

Missing values of covariates less than 5% were filled with multiple imputations.

b Data on alcohol drinking status, chronic kidney disease, and insufficient physical activity were not surveyed in 2019 which were excluded in the corresponding analysis.

## Supplemental Table 7: Rates of CRN and Its Components Across Subgroups of Patients with Chronic bronchitis^a^.

|  | Age <65 y | | | | Age≥65 y | | | |
| --- | --- | --- | --- | --- | --- | --- | --- | --- |
|  | (1) Skipped Medication Doses to Save Money | (2) Took Less Medicine to Save Money | (3) Delayed Filling a Prescription to Save Money | 1, 2, and/or 3=CRN | (1) Skipped Medication Doses to Save Money | (2) Took Less Medicine to Save Money | (3) Delayed Filling a Prescription to Save Money | 1, 2, and/or 3=CRN |
| Sample, n | 862 | 930 | 1098 | 1262 | 221 | 249 | 283 | 356 |
| Weighted sample, n (weighted %) | 764280(18.18) | 800013(19.03) | 953457(22.68) | 1083780(25.78) | 150690(8.53) | 165882(9.39) | 188848(10.69) | 245379(13.89) |
| Sex |  |  |  |  |  |  |  |  |
| Male | 16.82 | 17.55 | 21.18 | 24.05 | 6.65 | 6.83 | 7.77 | 9.99 |
|  | (14.04,20.04) | (14.75,20.76) | (18.31,24.37) | (21.02,27.37) | (4.93,8.92) | (4.98,9.29) | (5.83,10.29) | (7.72,12.82) |
| Female | 18.78 | 19.68 | 23.33 | 26.54 | 9.42 | 10.60 | 12.07 | 15.74 |
|  | (17.15,20.53) | (18.01,21.46) | (21.59,25.18) | (24.73,28.43) | (7.78,11.35) | (8.78,12.74) | (10.25,14.16) | (13.52,18.24) |
| Race/ethnicity |  |  |  |  |  |  |  |  |
| Non-Hispanic white | 17.66 | 18.30 | 21.92 | 24.84 | 7.85 | 8.64 | 10.03 | 13.07 |
|  | (15.96,19.50) | (16.64,20.09) | (20.15,23.80) | (23.00,26.78) | (6.49,9.48) | (7.08,10.50) | (8.49,11.81) | (11.19,15.21) |
| Hispanic | 20.66 | 21.77 | 28.14 | 30.61 | 11.08 | 11.04 | 11.53 | 14.29 |
|  | (15.80,26.54) | (16.94,27.51) | (22.99,33.94) | (25.34,33.25) | (6.20,19.05) | (6.16,19.01) | (7.04,18.33) | (8.82,22.31) |
| Non-Hispanic black | 20.09 | 21.20 | 24.79 | 28.84 | 13.71 | 13.99 | 17.30 | 20.89 |
|  | (16.74,23.92) | (17.67,25.23) | (21.11,28.87) | (24.81,33.25) | (9.65,19.11) | (9.98,19.28) | (12.78,22.99) | (15.97,26.84) |
| Non-Hispanic Asian | 11.24 | 14.66 | 9.78 | 15.35 | 6.99 | 16.48 | 6.99 | 16.48 |
|  | (5.08,23.03) | (7.63,26.31) | (4.10,21.57) | (8.19,26.93) | (1.72,24.38) | (6.51,35.86) | (1.72,24.38) | (6.51,35.86) |
| Region |  |  |  |  |  |  |  |  |
| Northeast | 14.09 | 12.35 | 15.16 | 17.31 | 7.83 | 9.98 | 9.44 | 15.60 |
|  | (11.20,17.58) | (9.61,15.74) | (12.21,18.67) | (14.16,21.00) | (5.07,11.91) | (6.06,16.01) | (6.45,13.60) | (10.66,22.25) |
| Midwest | 17.72 | 18.99 | 23.93 | 26.95 | 10.71 | 11.31 | 12.37 | 16.50 |
|  | (14.81,21.07) | (16.01,22.38) | (20.66,27.53) | (23.56,30.64) | (8.24,13.82) | (8.57,14.79) | (9.50,15.96) | (13.13,20.53) |
| South | 20.52 | 22.12 | 24.89 | 28.70 | 7.54 | 7.78 | 10.33 | 12.33 |
|  | (18.29,22.95) | (19.88,24.53) | (22.66,27.27) | (26.32,31.20) | (5.80,9.75) | (6.06,9.93) | (8.31,12.77) | (10.12,14.95) |
| West | 16.83 | 17.67 | 22.50 | 24.88 | 8.89 | 10.37 | 10.62 | 12.58 |
|  | (13.49,20.81) | (14.22,21.75) | (18.93,26.53) | (21.22,28.95) | (5.87,13.25) | (7.03,15.03) | (7.52,14.79) | (9.20,16.97) |
| Education level |  |  |  |  |  |  |  |  |
| Less than high school | 24.16 | 27.66 | 32.52 | 35.18 | 12.25 | 13.30 | 14.75 | 17.19 |
|  | (19.98,28.90) | (23.45,32.30) | (28.28,37.06) | (30.81,39.81) | (8.95,16.55) | (9.86,17.72) | (11.10,19.35) | (13.33,21.90) |
| High school graduate | 16.39 | 19.07 | 22.98 | 26.51 | 7.72 | 9.06 | 10.35 | 13.68 |
|  | (13.77,19.38) | (16.06,22.49) | (19.77,26.55) | (23.04,30.28) | (5.68,10.41) | (6.73,12.08) | (7.90,13.45) | (10.71,17.32) |
| College or above | 17.68 | 17.53 | 20.90 | 23.94 | 7.87 | 8.51 | 9.78 | 13.15 |
|  | (15.86,19.65) | (15.78,19.44) | (19.03,22.89) | (22.01,25.99) | (6.25,9.87) | (6.67,10.80) | (8.02,11.87) | (10.92,15.76) |
| Household income |  |  |  |  |  |  |  |  |
| Low | 24.21 | 25.63 | 30.14 | 33.52 | 13.35 | 14.47 | 16.75 | 21.19 |
|  | (21.76,26.84) | (23.16,28.27) | (27.63,32.78) | (30.95,36.19) | (10.99,16.12) | (11.98,17.36) | (14.14,19.73) | (18.29,24.41) |
| Middle | 16.98 | 18.17 | 23.42 | 26.73 | 7.80 | 8.87 | 8.98 | 13.06 |
|  | (14.31,20.04) | (15.42,21.28) | (20.38,26.75) | (23.48,30.24) | (5.70,10.59) | (6.27,12.40) | (6.91,11.60) | (10.02,16.84) |
| High | 9.68 | 9.45 | 10.70 | 13.37 | 3.84 | 3.77 | 4.94 | 5.95 |
|  | (7.57,12.29) | (7.43,11.96) | (8.58,13.26) | (10.99,16.18) | (2.12,6.83) | (2.01,6.96) | (2.87,8.38) | (3.69,9.47) |
| Insurance status |  |  |  |  |  |  |  |  |
| Public | 19.38 | 21.43 | 24.89 | 28.41 | 9.03 | 10.10 | 11.86 | 15.15 |
|  | (17.14,21.85) | (19.12,23.94) | (22.48,27.46) | (25.97,30.99) | (7.39,10.99) | (8.14,12.48) | (9.95,14.08) | (12.82,17.83) |
| Private | 16.40 | 16.48 | 20.41 | 23.15 | 7.67 | 8.35 | 9.13 | 12.05 |
|  | (14.71,18.23) | (14.77,18.36) | (18.60,22.36) | (21.23,25.19) | (5.86,9.98) | (6.42,10.79) | (7.14,11.61) | (9.75,14.81) |
| Uninsured | 45.81 | 44.64 | 43.96 | 49.37 | 23.98 | 18.84 | 16.84 | 30.66 |
|  | (29.45,63.13) | (28.44,62.05) | (27.86,61.43) | (32.80,66.08) | (9.15,49.69) | (6.27,44.61) | (5.60,40.87) | (13.00,56.67) |
| Smoking status |  |  |  |  |  |  |  |  |
| Never | 14.14 | 14.49 | 17.92 | 20.84 | 8.23 | 9.12 | 9.39 | 13.78 |
|  | (12.28,16.24) | (12.67,16.52) | (15.89,20.14) | (18.69,23.17) | (6.17,10.89) | (6.71,12.29) | (7.33,11.94) | (10.95,17.20) |
| Previous | 17.08 | 17.78 | 20.70 | 24.42 | 7.75 | 8.87 | 10.63 | 13.03 |
|  | (14.26,20.33) | (14.92,21.05) | (17.62,24.15) | (21.15,28.02) | (6.13,9.76) | (7.07,11.07) | (8.61,13.06) | (10.75,15.72) |
| Current | 23.91 | 25.44 | 29.83 | 32.76 | 11.79 | 11.75 | 14.15 | 16.97 |
|  | (21.04,27.03) | (22.59,28.51) | (26.75,33.10) | (29.65,36.04) | (8.40,16.32) | (8.40,16.21) | (10.49,18.82) | (12.93,21.95) |
| Alcohol drinking status^b^ |  |  |  |  |  |  |  |  |
| Never | 15.86 | 15.55 | 18.38 | 21.62 | 8.52 | 11.58 | 11.33 | 14.27 |
|  | (12.64,19.70) | (12.53,19.14) | (15.05,22.27) | (18.02,25.72) | (5.77,12.40) | (8.37,15.81) | (8.24,15.40) | (10.83,18.58) |
| Previous | 20.49 | 22.18 | 26.52 | 29.23 | 10.66 | 11.31 | 12.49 | 16.97 |
|  | (17.53,23.82) | (19.02,25.70) | (23.12,30.23) | (25.76,32.96) | (8.27,13.63) | (8.53,14.85) | (9.99,15.51) | (13.64,20.91) |
| Current | 17.93 | 18.76 | 22.39 | 25.59 | 7.23 | 7.16 | 9.32 | 11.84 |
|  | (16.05,19.97) | (16.85,20.82) | (20.43,24.49) | (23.53,27.78) | (5.67,9.19) | (5.53,9.22) | (7.51,11.52) | (9.80,14.25) |
| Comorbidities |  |  |  |  |  |  |  |  |
| Hypertension | 21.83 | 22.40 | 25.81 | 29.05 | 9.16 | 9.68 | 11.06 | 14.29 |
|  | (19.49,24.36) | (20.05,24.94) | (23.37,28.40) | (26.50,31.73) | (7.64,10.96) | (8.15,11.45) | (9.44,12.91) | (12.43,16.38) |
| High cholesterol | 20.63 | 21.09 | 25.33 | 28.47 | 9.63 | 9.72 | 12.24 | 15.65 |
|  | (18.27,23.21) | (18.78,23.61) | (22.86,27.96) | (25.90,31.18) | (8.00,11.55) | (8.06,11.67) | (10.38,14.39) | (13.52,18.05) |
| Coronary heart disease | 24.14 | 23.44 | 28.00 | 30.89 | 10.05 | 11.00 | 12.71 | 16.27 |
|  | (20.58,28.10) | (20.13,27.11) | (24.32,32.01) | (27.13,34.92) | (7.92,12.67) | (8.86,13.58) | (10.41,15.42) | (13.67,19.26) |
| Stroke | 25.20 | 27.60 | 29.17 | 31.23 | 10.53 | 9.47 | 11.88 | 14.11 |
|  | (19.63,31.74) | (22.06,33.92) | (23.52,35.56) | (25.48,37.61) | (7.08,15.39) | (6.29,14.01) | (8.26,16.79) | (10.17,19.24) |
| Asthma | 20.31 | 21.24 | 24.94 | 28.67 | 11.23 | 11.90 | 14.45 | 18.66 |
|  | (18.27,22.52) | (19.09,23.57) | (22.71,27.31) | (26.32,31.13) | (9.00,13.92) | (9.71,14.50) | (12.03,17.26) | (15.84,21.85) |
| Cancer | 22.67 | 22.91 | 28.08 | 31.97 | 6.21 | 6.93 | 8.29 | 10.91 |
|  | (18.51,27.45) | (18.93,27.44) | (23.55,33.11) | (27.13,37.23) | (4.53,8.47) | (5.14,9.28) | (6.33,10.81) | (8.61,13.74) |
| Diabetes | 24.28 | 24.80 | 28.32 | 32.30 | 11.04 | 11.79 | 14.68 | 17.10 |
|  | (20.51,28.51) | (21.13,28.88) | (24.54,32.43) | (28.31,36.57) | (8.51,14.20) | (9.09,15.15) | (11.70,18.26) | (13.89,20.89) |
| Chronic kidney disease^b^ | 29.47 | 32.34 | 36.96 | 39.90 | 5.98 | 7.85 | 10.24 | 12.38 |
|  | (22.00,38.23) | (24.82,40.90) | (29.13,45.55) | (31.91,48.47) | (3.85,9.17) | (5.21,11.66) | (6.92,14.89) | (8.69,17.34) |
| Insufficient physical activity^b^ | 17.18 | 17.66 | 21.75 | 25.34 | 8.14 | 8.15 | 9.72 | 12.07 |
|  | (15.21,19.33) | (15.66,19.86) | (19.59,24.07) | (23.07,27.75) | (6.22,10.58) | (6.27,10.54) | (7.69,12.21) | (9.78,14.81) |
| BMI |  |  |  |  |  |  |  |  |
| Normal(<25kg/㎡) | 17.40 | 19.16 | 22.06 | 24.29 | 6.64 | 9.20 | 8.67 | 11.67 |
|  | (13.97,21.46) | (15.65,23.26) | (18.26,26.40) | (20.50,28.54) | (4.49,9.73) | (6.21,13.43) | (6.24,11.94) | (8.41,15.98) |
| Overweight(25-30kg/㎡) | 17.10 | 18.29 | 22.45 | 25.79 | 6.73 | 8.44 | 10.07 | 12.33 |
|  | (14.56,19.99) | (15.60,21.34) | (19.64,25.53) | (22.64,29.21) | (5.01,8.98) | (6.38,11.10) | (7.83,12.87) | (9.81,15.37) |
| Obese(>30kg/㎡) | 18.90 | 19.31 | 22.97 | 26.23 | 10.86 | 10.17 | 12.23 | 16.23 |
|  | (17.02,20.93) | (17.43,21.35) | (20.95,25.11) | (24.17,28.40) | (8.76,13.40) | (8.20,12.56) | (10.09,14.75) | (13.68,19.16) |

Values indicate weighted percentage (95% CI), unless otherwise indicated. Abbreviations: CRN, cost-related medication nonadherence; BMI, body mass index.

a Data on Chronic bronchitis were not surveyed from 2019 to 2020.

Missing values of covariates less than 5% were filled with multiple imputations.

b Data on alcohol drinking status, chronic kidney disease, and insufficient physical activity were not surveyed in 2019 which were excluded in the corresponding analysis.

1. National Center For Health S. National center for health statistics; 年份;卷(期);
